# Supplementary material for: Prevalence and antibiotic resistance of Escherichia coli in urban and peri-urban garden ecosystems in Bangladesh
Source: PLoS One. 2025 Feb 6;20(2):e0315938. doi: 10.1371/journal.pone.0315938 (PMC11801607; doi:10.1371/journal.pone.0315938)
Supplement: S1 Table — (DOCX) [file pone.0315938.s001.docx]

**Table S1.** Sampling information of the study.

| **Sample Type** | **Dhaka North City Corporation (DNCC)** | | **Dhaka South City Corporation (DSCC)** | | **Gazipur City Corporation (GCC)** | |
| --- | --- | --- | --- | --- | --- | --- |
|  | **Rooftop** | **Surface** | **Rooftop** | **Surface** | **Rooftop** | **Surface** |
| Vegetables | 25 | 23 | 10 | 10 | 10 | 10 |
| Water | 10 | 8 | 2 | 2 | 3 | 2 |
| Soil | 8 | 11 | 3 | 3 | 2 | 3 |
| Sub Total | 43 | 42 | 15 | 15 | 15 | 15 |
| **Total** | **145** | | | | | |
