# Supplementary material for: Prevalence and antibiotic resistance of Escherichia coli in urban and peri-urban garden ecosystems in Bangladesh
Source: PLoS One. 2025 Feb 6;20(2):e0315938. doi: 10.1371/journal.pone.0315938 (PMC11801607; doi:10.1371/journal.pone.0315938)
Supplement: S2 Table — (DOCX) [file pone.0315938.s002.docx]

**Table S2.** Number of *E. coli* positive samples from the study areas.

| **Samples name** | **Dhaka North City Corporation (DNCC)** | | **Dhaka South City Corporation (DSCC)** | | **Gazipur City Corporation (GCC)** | |
| --- | --- | --- | --- | --- | --- | --- |
|  | **Rooftop** | **Surface** | **Rooftop** | **Surface** | **Rooftop** | **Surface** |
|  | *E. coli* positive samples /Collected samples | | | | | |
| Vegetables | 7/25 | 20/23 | 7/10 | 10/10 | 10/10 | 7/10 |
| Water | 0/10 | 1/8 | 1/2 | 1/2 | 2/3 | 0/2 |
| Soil | 2/8 | 8/11 | 3/3 | 2/3 | 2/2 | 2/3 |
| Sub Total | 9/43 | 29/42 | 11/15 | 13/15 | 14/15 | 9/15 |
| Total | 85/145 | | | | | |
| Overall prevalence of *E. coli* is 58.62% (95% CI 50.48 – 66.31%) | | | | | | |
