# Supplementary material for: Prevalence and antibiotic resistance of Escherichia coli in urban and peri-urban garden ecosystems in Bangladesh
Source: PLoS One. 2025 Feb 6;20(2):e0315938. doi: 10.1371/journal.pone.0315938 (PMC11801607; doi:10.1371/journal.pone.0315938)
Supplement: S3 Table — (DOCX) [file pone.0315938.s003.docx]

**Table S3.** Prevalence of *E. coli* in the study areas.

| Study areas | Prevalence (%) | 95% Confidence Interval (%) | *p-*value |
| --- | --- | --- | --- |
| Dhaka North City Corporation (DNCC) | 44.70 (38/85) | 34.59 – 55.28 | 0.015 |
| Dhaka South City Corporation (DSCC) | 80.0 (24/30) | 62.69 – 90.49 |  |
| Gazipur City Corporation (GCC) | 76.67 (23/30) | 59.07- 44.20 |  |
