# Supplementary material for: Prevalence and antibiotic resistance of Escherichia coli in urban and peri-urban garden ecosystems in Bangladesh
Source: PLoS One. 2025 Feb 6;20(2):e0315938. doi: 10.1371/journal.pone.0315938 (PMC11801607; doi:10.1371/journal.pone.0315938)
Supplement: S4 Table — (DOCX) [file pone.0315938.s004.docx]

**Table S4.** Prevalence of *E. coli* in the rooftop and surface gardens of the study areas.

| Study areas | Rooftop gardens | | | | Surface gardens | | |
| --- | --- | --- | --- | --- | --- | --- | --- |
|  | Prevalence (%) | 95% CI (%) | *p-*value | | Prevalence (%) | 95% CI (%) | *p-*value |
| DNCC | 20.93 (9/43) | 11.42 – 35.20 | 0.05 | | 69.04 (29/42) | 53.97 - 80.92 | 0.025 |
| DSCC | 73.33 (11/15) | 48.05 – 89.10 |  |  | 86.67 (13/15) | 62.12 - 97.63 |  |
| GCC | 93.33 (14/15) | 70.18 – 99.65 |  |  | 60.0 (9/15) | 35.74 – 80.17 |  |
| Overall prevalence = 46.57% | | | | Overall prevalence = 70.83% | | | |

Here- DNCC: Dhaka North City Corporation, DSCC: Dhaka South City Corporation, GCC: Gazipur City Corporation, CI: Confidence Interval.
