# Supplementary material for: Prevalence and antibiotic resistance of Escherichia coli in urban and peri-urban garden ecosystems in Bangladesh
Source: PLoS One. 2025 Feb 6;20(2):e0315938. doi: 10.1371/journal.pone.0315938 (PMC11801607; doi:10.1371/journal.pone.0315938)
Supplement: S5 Table — (DOCX) [file pone.0315938.s005.docx]

**Table S5**. Prevalence of *E. coli* in different samples of rooftop gardens.

| **Name of samples** | **Selected areas** | | | | | |
| --- | --- | --- | --- | --- | --- | --- |
|  | **Dhaka North City Corporation (DNCC)** | | **Dhaka South City Corporation (DSCC)** | | **Gazipur City Corporation (GCC)** | |
|  | **Prevalence (%)** | ***p-*value** | **Prevalence (%)** | ***p-*value** | **Prevalence (%)** | ***p-*value** |
| Vegetables | 28% (7/25) | 0.04 | 70% (7/10) | 0.05 | 100% (10/10) | 0.16 |
| Water | 0% (0/10) |  | 50% (1/2) |  | 66.67% (2/3) |  |
| Soil | 25% (2/8) |  | 100% (3/3) |  | 100% (2/2) |  |
