# Supplementary material for: Prevalence and antibiotic resistance of Escherichia coli in urban and peri-urban garden ecosystems in Bangladesh
Source: PLoS One. 2025 Feb 6;20(2):e0315938. doi: 10.1371/journal.pone.0315938 (PMC11801607; doi:10.1371/journal.pone.0315938)
Supplement: S6 Table — (DOCX) [file pone.0315938.s006.docx]

**Table S6.** Prevalence of *E. coli* in different samples of surface gardens.

| **Name of samples** | **Selected areas** | | | | | |
| --- | --- | --- | --- | --- | --- | --- |
|  | **Dhaka North City Corporation (DNCC)** | | **Dhaka South City Corporation (DSCC)** | | **Gazipur City Corporation (GCC)** | |
|  | **Prevalence (%)** | ***p-*value** | **Prevalence (%)** | **P value** | **Prevalence (%)** | ***p-*value** |
| Vegetables | 86.95% (20/23) | 0.05 | 100% (10/10) | 0.16 | 70% (7/10) | 0.05 |
| Water | 12.5% (1/8) |  | 50% (1/2) |  | 0% (0/2) |  |
| Soil | 72.73% (8/11) |  | 66.67% (2/3) |  | 66.67% (2/3) |  |
