# Supplementary figures and images for: Prevalence and antibiotic resistance of Escherichia coli in urban and peri-urban garden ecosystems in Bangladesh
Source: PLoS One. 2025 Feb 6;20(2):e0315938. doi: 10.1371/journal.pone.0315938 (PMC11801607; doi:10.1371/journal.pone.0315938)

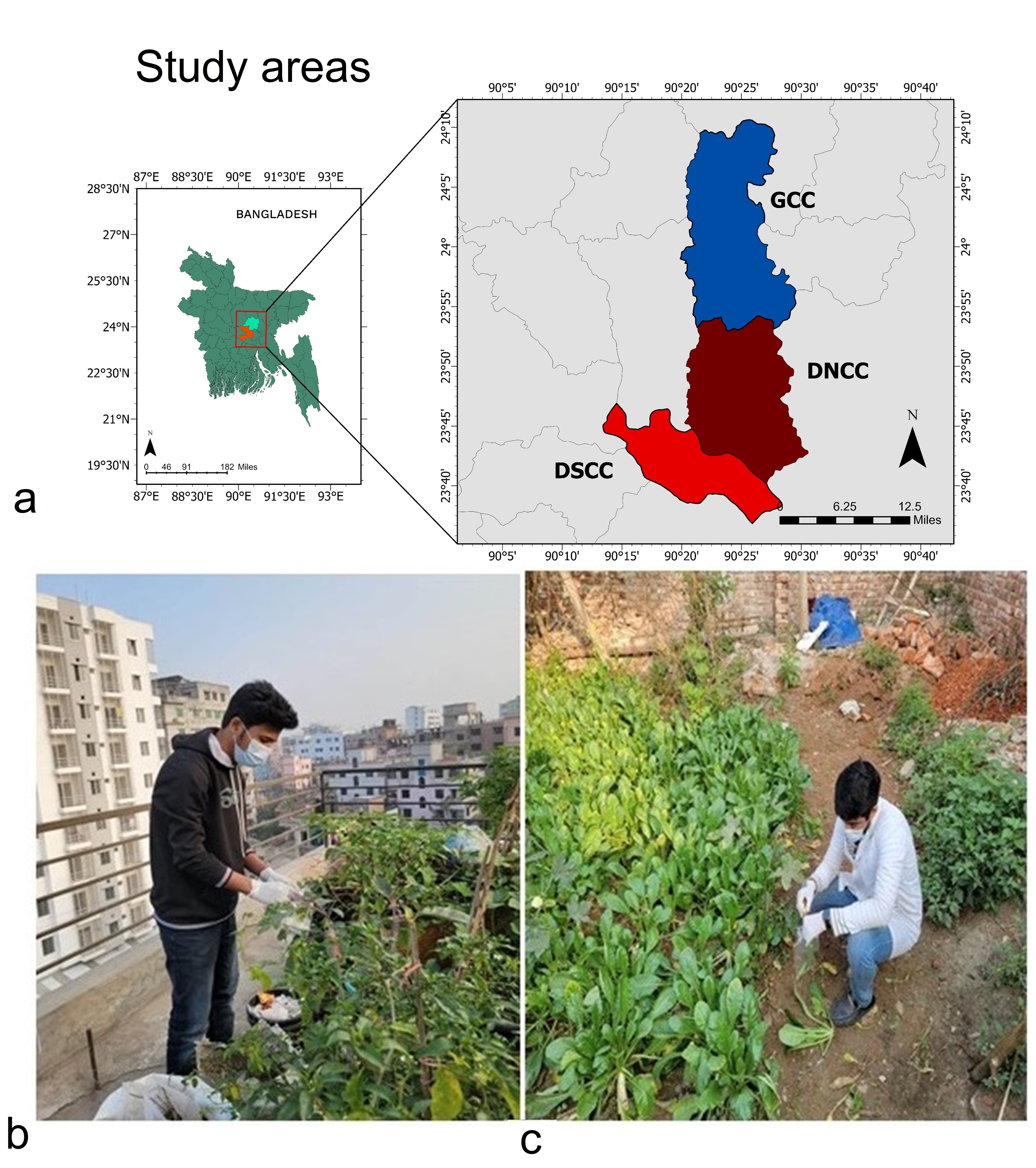

Supplement: S1 Fig — (a) Urban (Dhaka North City Corporation; DNCC and Dhaka South City Corporation; DSCC) and peri-urban (Gazipur City Corporation; GCC) areas of Bangladesh. (b) Rooftop gardens and (c) Surface gardens. (JPG) [file pone.0315938.s007.jpg]

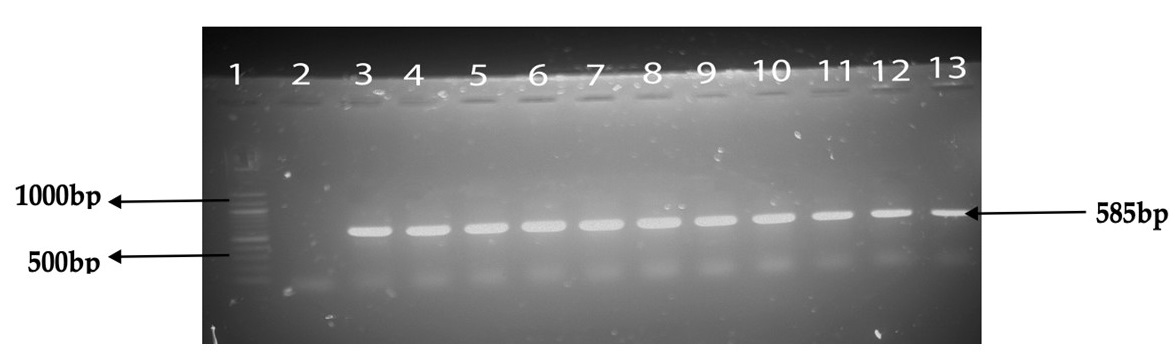

Supplement: S2 Fig — Lane 1: 1 kb DNA Marker; Lane 2: Negative control; Lane 3: Positive control; and Lane 4–13: Representative E. coli isolates. (JPG) [file pone.0315938.s008.jpg]

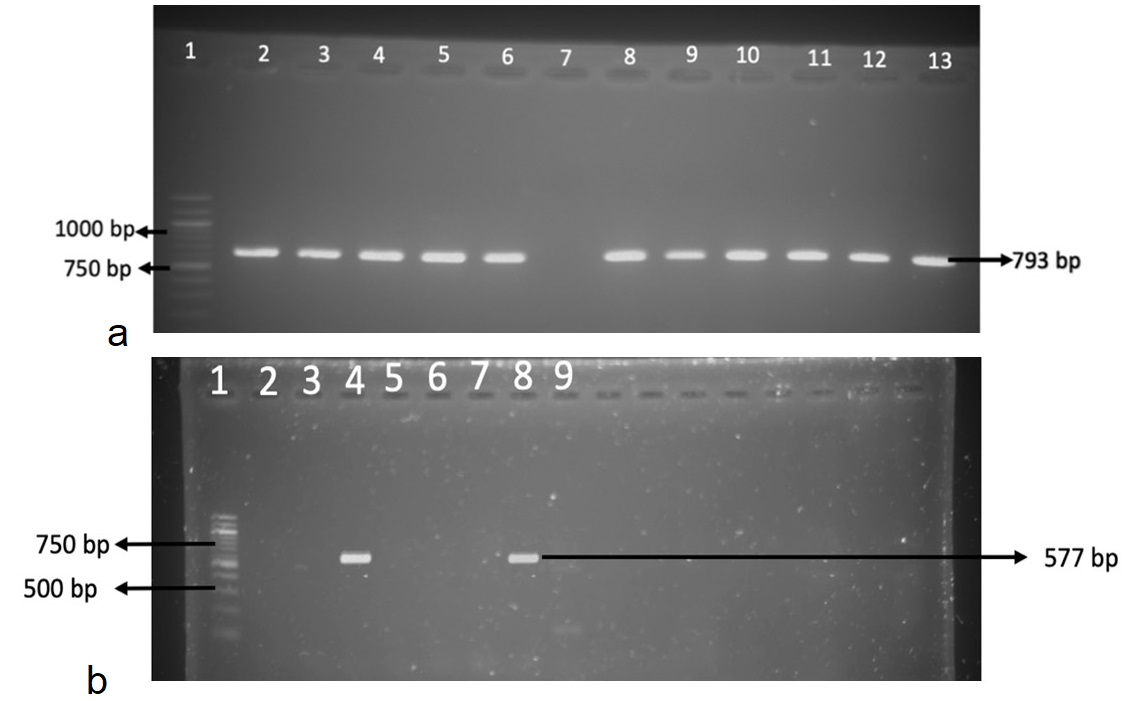

Supplement: S3 Fig — (a) PCR amplification of beta-lactamase-producing blaTEM gene in representative E. coli isolates. (b) PCR amplification of tetracycline resistance tetA gene in representative E. coli isolates. (JPG) [file pone.0315938.s009.jpg]
