# Supplementary material for: Prevalence and antibiotic resistance of Escherichia coli in urban and peri-urban garden ecosystems in Bangladesh
Source: PLoS One. 2025 Feb 6;20(2):e0315938. doi: 10.1371/journal.pone.0315938 (PMC11801607; doi:10.1371/journal.pone.0315938)
Supplement: Attachment — Submitted filename: renamed_a9e47.pdf [file pone.0315938.s010.pdf]

Point-by-point responses to the editor and reviewer's comments

Reviewer #1:

General Comment: The manuscript entitled: Prevalence and antibiotic resistance of Escherichia coli in urban and peri-urban garden ecosystems in Bangladesh" is a nicely written well-designed study. AMR is a major health problem across the globe. Here the authors have focused MDR *E. coli* in various gardening systems in Bangladesh. Methodologies are reproducible along with detailed results and discussion. Vegetables grown in rooftop gardens could be potential sources for MDR *E. coli*. As the authors have mentioned it is the first such study in Bangladesh describing MDR *E. coli* in rooftop vegetables, soil, and water in urban and peri-urban rooftop and surface gardens of Bangladesh. I believe the findings of the study could be considered in developing guidelines for better rooftop garden management in Bangladesh for better public health linked to MDR *E. coli*.

Our response: We do sincerely thank the reviewer for the encouraging our study and valuable suggestions for the writing improvement of the manuscript. You may kindly go through the revised manuscript.

Nevertheless, I have a few comments as follows:

Comment: Please write the function of malB gene used to detect *E. coli*.

Our response: Thank you for the comment. The *malB* gene is involved in the maltose and maltodextrin transport system in bacteria, particularly in *E. coli* [28]. The *malB* operon encodes components essential for the transport and metabolism of maltose and maltodextrins, which are polysaccharides derived from starch. This operon is part of the ATP-binding cassette (ABC) transporter family and includes genes like *malE*, *malF*, and *malG*, which encode for the maltose-binding protein (MalE) and the membrane components (MalF and MalG) that form the maltose transporter complex. You may kindly go through Lines 101-106 in the revised manuscript.

Name the vegetables under methodology section, scientific name…

Our response: Thank you. We have added the vegetable names as suggested. You may kindly go through Lines 128-132 in the revised manuscript.

What was the basis of selecting those antibiotics for the sensitivity test?

Our response: Thank you for the comment. As per Clinical Laboratory Standards Institute guideline 2023 (CLSI) M100 33rd Edition and antibiotics commonly used in Bangladesh we selected those antibiotics. Please refer to Lines 160-162 in the updated manuscript.

Please mention the year of CLSI in the methodology section, 2022?2023/2024??

Our response: Thank you for the comment. We have updated the manuscript accordingly. Please refer to Lines 160-162 in the updated manuscript.

What could be is the explanation for observing more E. coli in the surface garden than rooftop garden? Is it expected??

Our response: Thank you for the important comment. The elevated *E. coli* prevalence in surface gardens may stem from differences in soil type, irrigation, fertilization, and exposure to human or animal activity, which vary significantly from rooftop gardens [54, 55]. The role of soil as a primary reservoir for *E. coli* suggests its capacity to sustain and spread the bacterium to plants and ultimately to humans [56, 57]. Additionally, the use of unsanitized organic fertilizers or compost in surface gardens can introduce bacteria, while rooftop gardens exhibited no contamination in water samples, implying more controlled inputs. Please refer to Lines 328-338 in the updated manuscript.

Water samples from the rooftop gardens were found negative for E. coli, any speculation?

Our response: Thank you for the comment. Lower prevalence in water compared to vegetables may indicate dilution effects or sampling differences, while high soil prevalence reinforces soil's potential as an *E. coli* reservoir [57]. This may also be linked with the source of water e.g. deep tubewell water coming from the deep under surface free of contamination. Please refer to Lines 328-338 in the updated manuscript.

There are several tet family genes, why in this study only tetA gene primer was used for genotype.

Our response: Thank you for the comment. Yes, there are several tet family genes. tetA is more frequently found in tetracycline resistant so we used tetA primer/pcr only. In addition, we also do not have enough fund to do all the PCR.

Woo (2014) found a lower prevalence of blaTEM genes (3.6%) but a higher prevalence of tetA genes (10.7%), just the opposite of your study, what could be the reason??

Our response: Thank you for the comment. Its special and temporal variation. It may also be due to different level of use of antibiotics in these two study areas

Mention the major limitation of the study.

Our response: Thank you for the comment. We have mentioned it in the discussion now. . Please go through Lines 321-323 and 382-387 in the revised manuscript.

Reviewer ##2:

General Comment The manuscript entitled "Prevalence and antibiotic resistance of Escherichia coli in urban and peri-urban garden ecosystems in Bangladesh" describes the prevalence of Escherichia coli and its resistance status in raw vegetables, soil, and water samples collected from rooftop and surface gardens in urban and peri-urban areas of Bangladesh. From different reports, it is evident that family vegetable and fruit gardens on rooftops and surfaces are very common, particularly in city areas in Bangladesh. These gardens are a source of vegetables and a common area for family time. While gathering in gardens, family members, particularly children, sometimes eat vegetables and fruits without washing. However, the subject choice is appreciable as AMR in such a neglected area should be documented for policymakers. However, a few revisions and corrections will improve its quality. To me, the background of the study requires adding the concept of rooftop and surface gardens in city areas and explaining why city people are growing family gardens on rooftops and surfaces. In addition, please explain what agricultural practices are used in those gardens that could induce AMR in garden components.

Our response: We do sincerely thank the reviewer for the encouraging our study and valuable suggestions for the writing improvement of the manuscript. You may kindly go through the revised manuscript, especially Lines 317-323.

Line no. 118: Did the Authors use any sample size calculation formula? How did the Author finalize the 145 number of the sample? Any previous study in Bangladesh?

Our response: Thank you for your comment, and we apologize for the oversight. No, we did not perform a sample size analysis, as no previous studies exist in this area. This is the first preliminary survey comparing rooftop and surface garden samples.

Line no. 121: In Bangladesh, people generally cook vegetables at high temperatures rather than the salad types of vegetables. Therefore, it is essential to know the names and types of vegetables. In addition, the risk is different for raw and cooked vegetables. What is the source of the water sample? It is better to write a short paragraph about sample types, sources, etc.

Our response: Thank you very much for the concern. We have added the vegetable names as suggested. You may kindly go through Lines 128-132 in the revised manuscript.

Line no. 123, 140: Please make "E. coli" italic and search the whole manuscript and supplementary file for similar errors.

Our response: Thank you for the comment. We have checked and updated the manuscript. Please refer to Line 151 in the revised manuscript.

Line no. 131: Please avoid (.) color in writing.

Our response: Thank you for the comment. We have updated accordingly. Please refer to Line 142 in the revised manuscript.

Line no. 140-141: Based on the statement, positive control was used in PCR amplification. Please check the Fig. S2 and revise the figure legend.

Our response: Thank you for the comment. We have rewritten the legend and updated the supplementary file. Please refer to Fig. S2 in the revised version of the Supplementary Information File.

Line no. 161: Please mention the incubation period; generally, 16 hours is required to interpret the disc diffusion test.

Our response: Thank you for your comment. The isolated colonies were taken into 4 – 5 mL of nutrient broth for performing DDT. After preparing the broth cultures, isolates were incubated for 4 – 5 hrs at 37°C, and have now specified this in the manuscript. Please see Lines 166-168 in the revised manuscript.

Line no. 162: Please check the sentence – "multidrug resistance…."

Our response: Thank you for the comment. We have updated the sentence. Please see Line 173 in the revised manuscript.

Table 2: To me, in Pearson correlation analysis, when a variable is constant (100% or 0%), that variable is not computed. The prevalence of ampicillin resistance is 100%. Therefore, Pearson correlation for ampicillin is not possible. Please revise Table 2.

Our response: Thank you for the comment. Sorry for the mistake. We have removed the ampicillin data from the table as suggested. Please refer to Table 2 in the revised manuscript.

Table 3: Table 3 requires colossal revision. As per the statement, 54 isolates were randomly selected for the antimicrobial susceptibility test. Therefore, the number of isolates from different resistance patterns must be 54. However, it is only 17 from Table 3, column 4. Currently, among the 17 isolates, only eight are classified as MRD. Please add all 54 isolates in Table 3. Then, a different finding (maybe the pattern will be more, and the MDR percent will be changed) will be found. In addition, the heading of column 4 is "No. of MDR Isolates (%)." However, the patterns from rows 5 to 10 are not MDR by definition. Furthermore, the percentage in column 4 is incorrect, as they are not computed based on isolate number but antibiotic class. After the revision, there will be massive changes in results and discussion.

Our response: Thank you for your nice comment. We appreciate your concern about the table. Now we have reconsidered the table information and updated with the 54 isolates data. It was an unintentional mistake. Please refer to Table 3 in the revised manuscript.

Line no. 263-264: This data is not found in the Table 4. Please check for similar errors in the whole manuscript.

Our response: Thank you so much for your comment. We've checked the data and updated it. It should be Fig. 3 instead of Table 4.

Table 4: It is better to find the association of different tet genes in tetracycline resistance similarly to different bla genes for the beta-lactam antibiotics. However, this study does not have that design. Finding an association of tetA in some heterogeneous phenotypic resistance (ampicillin, ciprofloxacin, and others) is not useful and is the same for blaTEM. To establish such a relationship, please point them out in the discussion section with solid references.

Our response: Thank you for the comment. We agree with this comment, and now have deleted this table.

Line no. 267 -270: This information is not helpful. Among the eight tetracycline-resistant isolates, seven had blaTEM, which is only 15.9%, based on Table 4, and the Authors are presenting it as a low percentage of blaTEM in tetracycline resistance. However, it could be a maximum of 8 isolates, as the number of tetracycline resistance is 8. Then how could it be 100% or close to 100%? It is not possible. Therefore, this comparison is worthless. I suggest not to keep this table.

Our response: Thank you for the comment. We have deleted this table to avoid any confusion.

Line no. 265: Please make the gene name style uniform.

Our response: Thank you so much for your comment. Now, we've updated all the information. Please go through the revised manuscript.

Please check that the figure legends are missing and the figures are not self-explanatory.

Our response: Thank you so much for your comment. We have updated the legends of the figure and made them clearer to the reader.

Reviewer ###3:

1.The affiliation for co-author number 3 and 4 from Taiwan and Thailand should be completed like others.

Our response: Thank you for the comment. We have used the affiliation as the authors said so. This is how they use their name for publications. You may refer to https://doi.org/10.1007/s10661-024-12850-5

2. There were so many factors related to the difference between the rooftop and the surface gardens, thus, how different of soil, water and vegetable preparation among the 3 areas, Since this lack of the elementary information of these 3 areas of gardening for vegetable. Of course, that E. coli could be detected anywhere without any suspicious questions but the results seem not so benefit in term of garden management of vegetable for human consumption.

Our response: Thank you for your insightful comments. We acknowledge that understanding the differences in soil, water, and vegetable preparation among rooftop and surface gardens is crucial for interpreting our findings. While *E. coli* can be found in various environments, the lack of detailed information regarding gardening practices in the three areas limits the applicability of our findings for effective garden management. To enhance vegetable safety for human consumption, further research should focus on comparing soil quality, water sources, and preparation techniques. This would provide actionable insights for improving hygiene practices and mitigating contamination risks in both rooftop and surface gardens. To address your concerns, we plan to

incorporate a more detailed analysis of gardening practices in our future work. This will help establish clearer guidelines for improving garden management and ensuring the safety of vegetables for human consumption. We have revised the manuscript accordingly.

3. Please added the demographic information of DNCC, DSCC and GCC of how different of soil, water and vegetable growing in these 3 areas. Not just only details of 3 different location which show no meaning for E. coli isolation.

Our response: Thank you so much for your comment. We have updated the demographic information and samples name. You may kindly refer to Lines 118-124 in the updated manuscript.

4. The result shown that E. coli from rooftop garden was lower than surface garden, however, reader still do not know how difference of gardening management of these two kinds of garden and pricing of 2 different gardening reported in urban and peri-urban areas.

Our response: Thank you for the comment. The elevated *E. coli* prevalence in surface gardens may stem from differences in soil type, irrigation, fertilization, and exposure to human or animal activity, which vary significantly from rooftop gardens [54, 55]. The role of soil as a primary reservoir for *E. coli* suggests its capacity to sustain and spread the bacterium to plants and ultimately to humans [56, 57]. Additionally, the use of unsanitized organic fertilizers or compost in surface gardens can introduce bacteria, while rooftop gardens exhibited no contamination in water samples, implying more controlled inputs. Lower prevalence in water compared to vegetables may indicate dilution effects or sampling differences, while high soil prevalence reinforces soil's potential as an *E. coli* reservoir [57]. This underscores the public health significance of soil in urban and rooftop gardens, where contaminated soil can directly impact food safety and increase the risk of *E. coli*-related infections. You may kindly go through Lines 328-338 in the revised manuscript.

5. How come only AMR genes of blaTEM and tetA were studied? How about the others? This is the limitation of genotypic study in this research work. Where is malB?

Our response: Thank you for your comment. We acknowledge that a limitation of our study is the lack of funding, which prevented us from conducting PCR for all resistance genes. The *malB* gene used in our study was specifically for the detection of *E. coli*, rather than for identifying resistance genes. We previously performed PCR for the *malB* gene to confirm the isolates as *E. coli*. You may kindly go through the revised manuscript.

6. This study and the conclusion lacks of management issue after finding the E. coli contamination in Vegetable, of which the contamination normally found in fresh vegetable. Just only mentioned in line between 307-309 is too few.

Our response: Thank you very much for this concern. We've now elaborately discussed this section. You may kindly go through Lines 397-402 in the revised manuscript.

7. Line 369-371: the recommendation should be more strictly applicable rather than only using potable water to clean the rooftop garden. Any other application for the surface garden should be done as well??

Our response: Thank you for the important comment. Now updated: we have mentioned "public awareness" under this section. You may kindly go through Lines 397-402 in the revised manuscript.

6. Why didn't author mentioned about malB gene in the genotypic study? It didn't mention at all so please add details inside. Thank you for the comment. malB gene was uses to conform the isolation of E. coli, (not for any genotyping) so we did not focused on malB gene genotyping.

Our response: Thank you for the comment. The *malB* gene is involved in the maltose and maltodextrin transport system in bacteria, particularly in *E. coli* [28]. The *malB* operon encodes components essential for the transport and metabolism of maltose and maltodextrins, which are polysaccharides derived from starch. This operon is part of the ATP-binding cassette (ABC) transporter family and includes genes like *malE*, *malF*, and *malG*, which encode for the maltose-binding protein (MalE) and the membrane components (MalF and MalG) that form the maltose transporter complex. You may kindly go through Lines 101-106 in the revised manuscript.

Reviewer ####4:

1. Authors are requested to update the reference list and cite with some recent articles.

Our response: We do sincerely thank the reviewer for the encouraging our study and valuable suggestions for the writing improvement of the manuscript. We've updated the reference section by citing recent articles. Please refer to the updated manuscript.

2. Line 140. E. coli should be in Italic. Please check this throughout the manuscript.

Our response: Thank you for your comment. We have updated this information. Please see Line 151 in the revised manuscript.

3. Line 111 Cross-sectional studies of what?? Make it clear

Our response: Thank you for the feedback. We have removed the term "cross-sectional studies" to avoid any confusion. Please refer to Lines 116-118 in the revised manuscript.

4. What are the microflora of rhizospheric soils? Please identify the soil microflora of each sampling sites and including the findings in the revision.

Our response: Thank you for the comment. It was not the objective of this study to focus on microflora of rhizospheric soils, so it is out of scope of this study to focus on microflora of rhizospheric soils.

5. Is there is any horizontal gene transfer between E. coli with plant associated bacteria?

Our response: Thank you for the valuable comment. We are not aware of any such event.

6. Authors are requested to draw a scheme how E. coli adapts to plants defense molecules viz salicylic and jasmonic acid?. How plants induce selection for resistant E. coli?

Our response: Thank you for the comment. It is beyond the scope of this study so not possible to address.

7. Authors may also check the presence of AvrE, HopZ proteins in any plant system to understand how E. coli-plant interactions.

Our response: Thank you for the comment. It was not part of this study to focus on any proteins, so did not focus on these AvrE, HopZ proteins.

8. Picture quality and presentation is poor. Please increase the dpi at least 300 dpi.

Our response: We have revised and updated the resolution of the figures. Please refer to the JPEG format of the images in the revised submission.

9. Why GCC and DSCC rooftop have prevalence of E. coli >70? Explain

Our response: Thank you for the comment. Probably due to any sort of unknown contamination of these gardens??
